# No evidence for social factors in the overestimation of individuals from minority groups

**DOI:** 10.1073/pnas.2214740119

**Published:** 2022-11-07

**Authors:** Surya Gayet, Andre Sahakian, Chris L. E. Paffen, Stefan Van der Stigchel

**Affiliations:** ^a^Experimental Psychology, Helmholtz Institute, Utrecht University, Heidelberglaan 1, 3584 CS Utrecht, The Netherlands

It has been well-established that observers tend to overestimate the prevalence of underrepresented samples in a population, such as a minority of blue marbles among a majority of red marbles (1–5). A recently published article (6) replicates this finding using faces of ethnic minority and ethnic nonminority groups but reaches the additional conclusion that the overestimation of underrepresented samples is enhanced by social knowledge; specifically, the article concludes that the prevalence of underrepresented samples is overestimated even more when these samples are known to be ethnic minorities in the observers’ society.

This intriguing conclusion was based exclusively on their Experiment 5, in which (White American) observers estimated the proportion of African and White American faces within stimulus displays. African American faces could either be underrepresented or overrepresented in the stimulus displays. The data showed that the overestimation—of faces that were underrepresented in the display—was actually larger when African American faces, rather than White American faces, were underrepresented. This result is interpreted as evidence that social knowledge about African Americans being a minority in society contributed to the overestimation of African American faces in the display and is presented as the main finding of the article.

Here, we raise the concern that proportion estimates of different stimuli can be influenced by inherent differences in visual characteristics between these stimuli, irrespective of the social content of the stimuli. In the target article, White and African American faces differed in luminance and thereby varied in discriminability from the white stimulus background; this is likely to cause an asymmetry in proportion estimates. Without properly controlling the discriminability of the faces from the background, conclusions about nonvisual (e.g., social) factors are not warranted.

To illustrate this concern, we conducted two replication studies of their Experiment 5, one in which the background was white and one in which it was black (keeping all other aspects the same as in the original study; total *n* = 199; Fig. 1). Our data reveal that the critical interaction reported in the target article (i.e., between ethnicity of the reported face and ethnicity of the minority group) indeed depends on the color of the background (three-way interaction, *F*_1,185_ = 18.35, *P* < 0.001). Moreover, in a separate set of experiments (total *n* = 195; Fig. 2), we obtain this same pattern of results when replacing White and African American faces with light and dark gray circles, respectively (both when measuring proportions per trial, *F*_1,187_ = 15.38, *P* < 0.001, or over the entire experiment at once, *F*_1,187_ = 5.43, *P* < 0.05). Thus, an asymmetry in minority overestimation between stimulus conditions, akin to the one observed in the target article, can be obtained with stimuli that carry no social information whatsoever. For full methods and materials see https://osf.io/pkscm/.

**Fig. 1.**
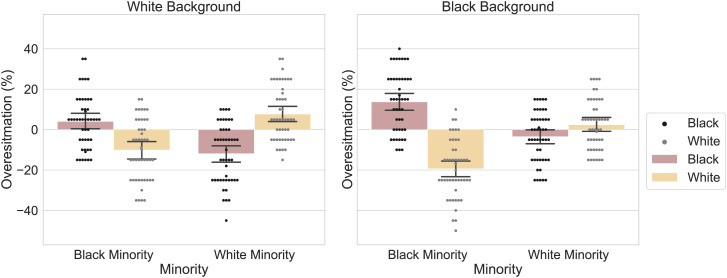
Replication of Experiment 5 from Kardosh et al. (6), on two differently colored backgrounds: white (*Left*) or black (*Right*). Participants estimated the proportion of White (light gray dots) and African American faces (black dots) in Black-minority or White-minority stimulus displays.

**Fig. 2.**
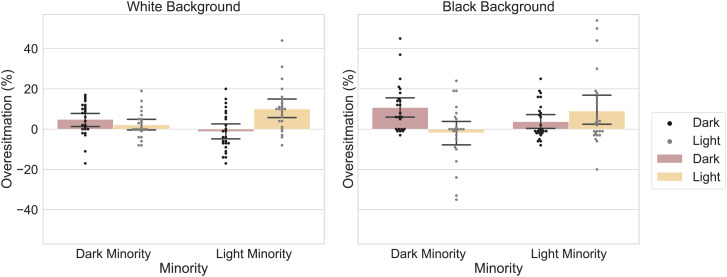
Same as Fig. 1, but now African and White American faces were replaced by dark gray and light gray discs (depicted as black and light gray dots in the graph, respectively).

Taken together, we deem it unjustified to attribute modulations of minority overestimation to social knowledge, when 1) the direction of this modulation can be reversed by altering basic visual characteristics of the display and 2) similar modulations can be obtained with stimuli that are devoid of social information.

## References

[1] I. Erev, T. S. Wallsten, D. V. Budescu, Simultaneous over- and underconfidence: The role of error in judgment processes. Psychol. Rev. 101, 519–527 (1994).

[2] R. T. Zacks, L. Hasher, Frequency processing: A twenty-five year perspective. Freq. Process. Cogn. 6, 21–36 (2002).

[3] W. K. Viscusi. Smoking: Making the Risky Decision (Oxford University Press, 1992).

[4] W. K. Viscusi, J. K. Hakes, Risk beliefs and smoking behavior. Econ. Inq. 46, 45–59 (2008).

[5] B. Fischhoff , Teen expectations for significant life events. Public Opin. Q. 64, 189–205 (2000). 10984333 10.1086/317762

[6] R. Kardosh, A. Y. Sklar, A. Goldstein, Y. Pertzov, R. R. Hassin, Minority salience and the overestimation of individuals from minority groups in perception and memory. Proc. Natl. Acad. Sci. U.S.A. 119, e2116884119 (2022). 35286213 10.1073/pnas.2116884119 PMC8944588

